# Characteristics of children with cat sensitivity: a prospective cross-sectional study

**DOI:** 10.55730/1300-0144.5592

**Published:** 2022-12-26

**Authors:** Ezgi ULUSOY SEVERCAN, Nevzat BAŞKAYA, Ayşegül ERTUĞRUL, Zeynep ŞENGÜL EMEKSİZ, İlknur BOSTANCI

**Affiliations:** Department of Pediatric Immunology and Allergy, University of Health Sciences, Dr. Sami Ulus Maternity and Children Training and Research Hospital, Ankara, Turkey

**Keywords:** Allergic rhinitis, asthma, cat, cat sensitivity, total IgE

## Abstract

**Background/aim:**

Animal dander is one of the most common respiratory allergens in children, and there is evidence that cat sensitivity is a risk factor for asthma and allergic rhinitis. In this study, it was aimed to evaluate children with cat sensitivity and to identify their demographic and clinical characteristics.

**Materials and methods:**

Patients who were found to be sensitive to cats following skin prick tests performed in our allergy clinic over a period of one year (and two control groups), were included in the study. Patients in the study and control groups filled in a questionnaire including demographic and clinical characteristics.

**Results:**

The prevalence of cat sensitivity in our allergy clinic was 6% (182/3033). The most common diagnoses in patients were 41.8% allergic rhinitis, 25.8% asthma, and 13.2% allergic rhinitis + asthma. Allergic rhinitis symptoms were the most prevalent symptom associated with cat contact (29.4%), whereas 28% of the patients were asymptomatic. Only 17.3% had a cat at home and 13.4% had cat exposure apart from home but having a cat at home was significantly higher than the control groups (p < 0.05). Eosinophilia was present in 54.6% of the patients, and 17.3% had blood tIgE levels of >1000 IU/mL. Eosinophilia and tIgE levels were significantly higher than both control groups (p < 0.05).

**Conclusion:**

Cat ownership can affect the development of cat sensitivity but the majority of patients with cat sensitivity are not cat owners. Elevated tIgE levels (> 1000 IU/mL) may be associated with cat sensitivity, these patients should be evaluated for cat sensitivity, even if they do not report symptoms with cat contact.

## 1. Introduction

Allergic diseases especially allergic rhinoconjunctivitis and asthma have a high prevalence all over the world and they appear to be an important public health problem [1]. Animal dander is one of the most common respiratory allergens in children and there is evidence that cat sensitivity is a risk factor for asthma and allergic rhinitis [1–5]. Cat sensitization prevalence varies worldwide but it has increased in recent decades [6]. The range differs from 4.7%–12.1% in children in Europe [6,7]. Children in both cat- and noncat-owning houses have been shown to have cat allergies. Children can also be exposed to cat allergens in schools and other public places [3,6,8]. As an initial measure, skin prick tests with standardized extracts can be used to identify cat sensitivity in patients with a medical history. When a patient’s history and skin prick tests are inconclusive, a serum-specific IgE test is advised since it has poor specificity but high sensitivity [9]. Fel d 1 is the major cat allergen found in more than 90% of cat allergenic patients [3].

There is limited data regarding cat-sensitive children’s characteristics in the literature. In this study, it was aimed to evaluate patients with cat sensitivity and to identify their demographics and clinical characteristics.

## 2. Materials and methods

This study was conducted at Dr. Sami Ulus Maternity and Children Training and Research Hospital, in Ankara, Turkey, and was designed as a prospective cross-sectional study. The inclusion and exclusion criteria for study patients were:

Inclusion criteria: 1. Patients who underwent a skin prick test for any indication (asthma, wheezy child, allergic rhinitis, atopic dermatitis, urticaria, etc.) and were found to be sensitive to cats in skin prick tests in the allergy clinic in January–December 2019 period were included in the study.

Exclusion criteria: 1. Patients who did not want to participate in the study.

**Figure 1:**
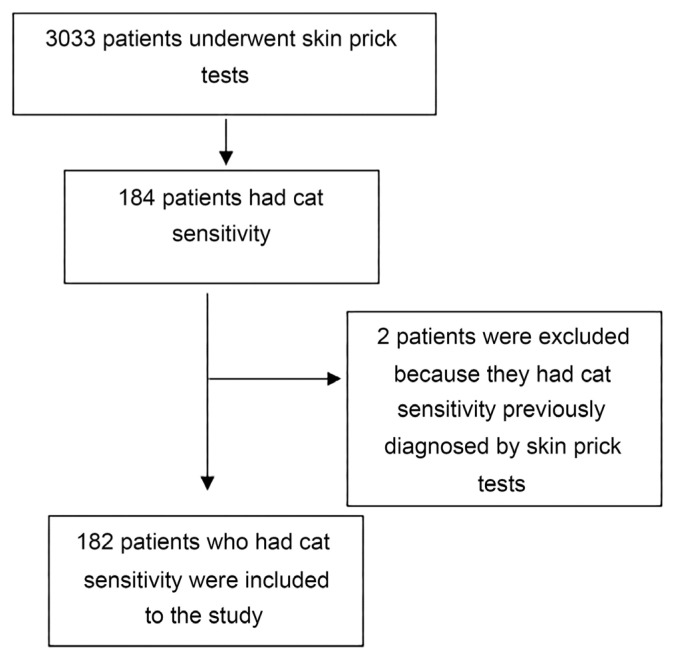
Flowchart for inclusion of patients.

2. Patients who had accompanying parasitosis such as *Enterobius vermicularis, Ascaris lumbricoides, Taenia saginata, etc*.

3. Patients who had chronic diseases other than allergies (rheumatoid arthritis, cystic fibrosis, immunodeficiencies, epilepsy, etc).

4. Patients with cat sensitivity previously diagnosed by skin prick tests were excluded (Figure 1).

Two control groups were also selected randomly from patients on whom skin prick tests had been performed during the same period in our clinic for any indication. One control group included patients who did not have any allergen sensitization in the skin prick tests. The second control group included patients with at least one aeroallergen sensitization other than cats. Two different control groups were formed in order to evaluate the differences from the population with no sensitivity and also to evaluate the differences from between patients with sensitivities other than cats. In particular, it is aimed to determine the characteristics that may arise from the cat independently of other sensitivities.

Skin prick tests were performed with a standard panel consisting of 9 allergen extracts: grass mix, cereal mix, tree mix, *felis domesticus, canis familiaris, dermatophagoides pterogyneous (DP), alternaria alternata (AA), cupressus sempervirens (CS), artemisia vulgaris (AV)*, and histamine (10 mg/mL of histamine phosphate) as positive and 0.9% sterile saline as negative controls. Skin prick tests were evaluated 15–20 min after application and were considered positive if the mean wheal diameter was ≥3 mm. Cat sensitivity was defined as a positive skin prick test for *felis domesticus*. Patients in the study and control groups filled in a questionnaire including demographics and clinical characteristics, during their outpatient visits. Eosinophil counts and serum total IgE (tIgE) levels were also investigated. Levels of ≥ 4% were accepted as eosinophilia.

The study was approved by the ethics committee of Etlik Zübeyde Hanım Maternity and Women’s Health Training and Research Hospital (number: 2020/156.). Informed consent was obtained from patients.

Data analyses were performed using the Statistical Package for Social Sciences version 22.0. All data were presented as percentages or mean ± standard deviation (SD). Comparisons between groups were analyzed using Pearson’s chi-squared test. P < 0.05 was taken as a criterion for statistically significant differences.

## 3. Results

The number of children who had a skin prick test at our allergy clinic between January–December 2019 were 3033, 182 of whom were sensitive to cats (6%). The demographic characteristics of children was shown in Table.

### 3.1. Diagnoses of the patients

**Figure 2:**
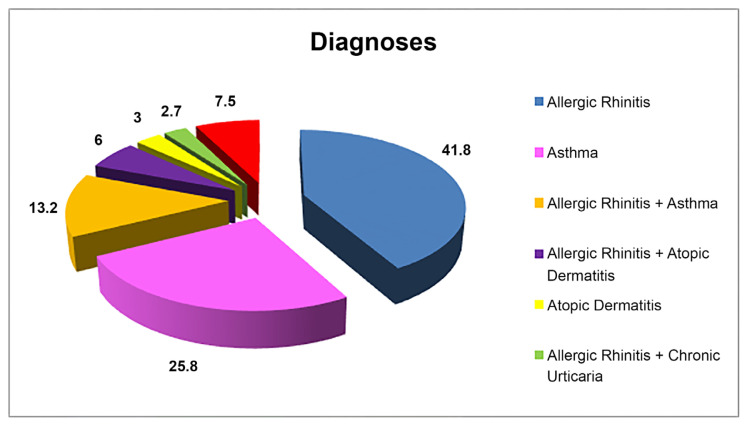
Diagnosis of the patients.

The most common diagnoses in the patients were, (41.8%) allergic rhinitis, (25.8%) asthma, (13.2%) allergic rhinitis + asthma, (6%) allergic rhinitis + atopic dermatitis (Figure 2).

### 3.2. Cat ownership

Only 17.3% had a cat at home and 13.4% had cat exposure outside of the home (neighbors, relatives, friends, etc.) but the prevalence of having a cat at home was significantly higher than in the two control groups (p: 0.001, p: 0.003). There was no statistically significant correlation between cat ownership and being asymptomatic with cat contact (p: 0.7).

### 3.3. Symptoms with cat contact

The most common symptom with cat contact was allergic rhinitis symptoms (runny nose, itching, sneezing) (29.4%). Asthma symptoms (wheezing, coughing) were 7.8%, urticaria/angioedema 3.3%. As a result of having no contact with cats, 25% of people were unsure if they had any symptoms, while 28% were asymptomatic. One patient (0.5%) developed anaphylaxis with cat dander exposure.

### 3.4. Skin prick test and laboratory results

The prevalence of children who were only sensitive to cats in skin prick tests was 13.7% (n: 25). There was no significant difference between patients with/without a cat at home in terms of only cat sensitivity (p: 0.49). Dog sensitivity was present in 11.5% (n: 21) children. The most common accompanying aeroallergen sensitivities were 6 grass types (59.8%), 4 cereals (57.1%), DP (20.3%), CS (13,3%), and AA (12.2%). Eosinophilia was present in 54.6% of the patients, and 17.3% had blood tIgE levels of >1000 IU/mL. The percentages of the patients with eosinophilia and high tIgE levels were significantly higher than in the control groups (p: 0.009, p: 0.001 and p: 0.006, p: 0.018) compared to the cat-sensitive group.

## 4. Discussion

In this study, cat sensitivity in children was assessed, and their characteristics were compared with those of sensitive and nonsensitive control groups. Cat sensitivity prevalence in this study group was found 6%. Similar to our finding Buyuk Yaytokgil et al. [10] found the prevalence of cat sensitivity to be 7.6% in their clinic. They also mentioned that the cat sensitization rate was higher during the pandemic. The most frequent diagnosis among these individuals, with or without comorbid additional allergic disorders, was allergic rhinitis (66.2%). In the study of Buyuk Yaytokgil et al. [10], also the most common diagnosis was allergic rhinitis (76%), but their anaphylaxis rate with cat exposure was high at 9% whereas it was 0.5% in our study.

The most common symptoms with cat contact were also allergic rhinitis symptoms such as runny nose, itching, and sneezing (29.4%) and 28% of the patients did not have any symptoms with cat contact. Although the prevalence of asymptomatic patients with cat sensitivity or the typical symptoms associated with cat encounters are not well-documented in the literature, the rate of being asymptomatic in the study of Buyuk Yaytokgil et al. [10] was found to be much higher (55.5%) than our study. This may be because, in 25% of the patients, it cannot be determined whether they are symptomatic by cat contact, as they do not have contact with the cat or cannot remember it. Kang et al. [11] mentioned that cat dander spIgE level might be useful for the exclusion of allergic symptoms related to pet exposure. In our study, we did not evaluated cat dander spIgE.

While the prevalence of cat ownership was 17.3%, the prevalence of those who did not own a cat but were exposed to cats due to friends, neighbors, relatives, etc. was 13.4 %. As a result, two third of the patients had no known cat exposure. This result was compatible with the knowledge that cat allergens can also be found in homes without cats [8,12–14]. In addition, this meant individuals’ exposure not only in the home environment but also in public spaces, parks, and streets could affect the development of cat sensitivity.

Most of the patients were multi-sensitive (86.2%) and there was no relationship between cat ownership and those who were sensitive only to cats and not to any other allergens. This showed that only cat sensitivity was not also strictly related to cat ownership. The most common accompanying aeroallergen was grass. Bostan et al. [15] stated in their study that *Timothy* allergy may play a role in the development of cat sensitivity. The fact that the most common allergen accompanying our patients was 6 grass types may be due to this relationship.

While some studies have found a positive correlation between cat ownership and cat sensitivity, some found a negative correlation and some did not find any correlation [16–22]. In this study, cat ownership in the study group was significantly higher than in either control group. Bostan et al. [15] also found cat ownership as a risk factor for cat sensitivity. In their study where they analysed dust samples of individuals’ houses, Custovic et al. [23] found that the prevalence of sensitization to cats was less in the lowest and highest cat allergen exposure groups and it was significantly more in the medium exposure group. We did not evaluate the level of exposure in this study.

We know that eosinophilia and high total IgE levels can be seen in patients with allergic sensitization. However, the eosinophilia and tIgE levels of the cat-sensitive group in this study were significantly higher than those of not only the nonsensitive control group but also the sensitive control group. This showed that cat allergens increase eosinophil levels and tIgE levels more than other allergens. It is noteworthy that especially in patients with cat sensitivity, the cases where tIgE level of >1000 IU/mL exceeded those in the control groups.

### 4.1. Study strengths and limitations

To the best of the author’s knowledge, this is the first study evaluating the characteristics of children with cat sensitivity and comparing them with sensitive and nonsensitive control groups. Two different control groups were formed in order to evaluate the differences from the population with no sensitivity and also to evaluate the differences between patients with sensitivities other than cats. The aim was to determine the differences that may arise with cat sensitivity, unlike other sensitivities. This is the strength of the study.

We did not measure cat allergens in patients’ houses. This may be a limitation. We did not perform a challenge test with the cat and we did not evaluate cat dander-specific IgE levels. Symptoms caused by cat contacts were based on the patients’ statements. Additionally, because there was no cat interaction or the patients could not recall, we were unable to determine in 25% of the patients if their symptoms were related to cat contact. We found a cat sensitivity prevalence of 6% in our allergy clinic. This only represents the prevalence among patients in an allergy clinic, not the population.

In conclusion, the most common symptoms with cat sensitivity are allergic rhinitis symptoms but one-quarter of the patients are asymptomatic. Although cat ownership is a risk factor for cat sensitivity, it is not necessary to have a cat to develop cat sensitivity. Elevated tIgE levels of > 1000 IU/mL may be associated with cat sensitivity. Even if these individuals do not associate their symptoms with cat contact, cat sensitivity should still be examined as a potential cause of the elevated tIgE levels.

## Figures and Tables

**Table:** Demographic characteristics of the patients with cat sensitivity.

Characteristics	

Gender	Male: 66.5% (n: 121)
	Female: 33.5% (n: 61)

Type of birth	Vajinal birth 46.1%
	Cesarean section 53.9%

Maturity	Preterm: 1.1%
	Term: 97.3 %
	Postterm: 1.6%
Consangunity	15.9%

Average age (months)	124 ± 51

Average age of symptom onset (months)	85 ± 51

Family history of atopy	45.6% (n: 83)

Family history of cat sensitivity	2.1%

Living area	City center: 92.9% (n: 169)
	Town: 6.6 % (n: 12)
	Village: 0.5 % (n: 1)

Accomodation	Apartment: 93.4% (n: 170)
	Single house with garden: 6.6% (n: 12)

Cat ownership	17.3% (n: 31)

Cat exposure outside of home	13.4% (n: 24)
